# Long COVID exhibits clinically distinct phenotypes at 3–6 months post-SARS-CoV-2 infection: results from the P4O2 consortium

**DOI:** 10.1136/bmjresp-2023-001907

**Published:** 2024-04-24

**Authors:** Jelle M Blankestijn, Mahmoud I Abdel-Aziz, Nadia Baalbaki, Somayeh Bazdar, Inés Beekers, Rosanne J H C G Beijers, Lizan D Bloemsma, Merel E B Cornelissen, Debbie Gach, Laura Houweling, Sebastiaan Holverda, John J L Jacobs, Reneé Jonker, Ivo van der Lee, Paulien M A Linders, Firdaus A A Mohamed Hoesein, Lieke C E Noij, Esther J Nossent, Marianne A van de Pol, Daphne W Schaminee, Annemie M W J Schols, Lisanne T Schuurman, Brigitte Sondermeijer, J J Miranda Geelhoed, Joop P van den Bergh, Els J M Weersink, Yolanda de Wit-van Wijck, Anke H Maitland-van der Zee

**Affiliations:** 1 Department of Pulmonary Medicine, Amsterdam UMC Locatie AMC, Amsterdam, The Netherlands; 2 Department of Clinical Pharmacy, Assiut University Faculty of Pharmacy, Assiut, Egypt; 3 ORTEC, Zoetermeer, Zuid-Holland, The Netherlands; 4 Department of Respiratory Medicine, NUTRIM School of Nutrition and Translational Research in Metabolism, Maastricht University Medical Centre+, Maastricht, The Netherlands; 5 Universiteit Maastricht School of Nutrition and Translational Research in Metabolism, Maastricht, The Netherlands; 6 Department of Environmental Epidemiology, Utrecht University Institute for Risk Assessment Sciences, Utrecht, The Netherlands; 7 Longfonds, Amersfoort, Utrecht, The Netherlands; 8 Department of Pulmonology, Spaarne Gasthuis, Haarlem, The Netherlands; 9 Department of Radiology, University Medical Center Utrecht and Utrecht University, Utrecht, The Netherlands; 10 Department of Respiratory Medicine, NUTRIM School for Nutrition, Toxicology and Metabolism, Maastricht University Medical Center+, Maastricht, The Netherlands; 11 Department of Respiratory Medicine, Leiden University Medical Center, Leiden, The Netherlands; 12 Department of Internal Medicine, VieCuri Medical Centre, Venlo, The Netherlands; 13 Department of Respiratory Medicine, Amsterdam UMC, Amsterdam, The Netherlands; 14 Department of Pediatric Respiratory Medicine, Emma Childrens' Hospital UMC, Amsterdam, The Netherlands

**Keywords:** COVID-19

## Abstract

**Background:**

Four months after SARS-CoV-2 infection, 22%–50% of COVID-19 patients still experience complaints. Long COVID is a heterogeneous disease and finding subtypes could aid in optimising and developing treatment for the individual patient.

**Methods:**

Data were collected from 95 patients in the P4O2 COVID-19 cohort at 3–6 months after infection. Unsupervised hierarchical clustering was performed on patient characteristics, characteristics from acute SARS-CoV-2 infection, long COVID symptom data, lung function and questionnaires describing the impact and severity of long COVID. To assess robustness, partitioning around medoids was used as alternative clustering.

**Results:**

Three distinct clusters of patients with long COVID were revealed. Cluster 1 (44%) represented predominantly female patients (93%) with pre-existing asthma and suffered from a median of four symptom categories, including fatigue and respiratory and neurological symptoms. They showed a milder SARS-CoV-2 infection. Cluster 2 (38%) consisted of predominantly male patients (83%) with cardiovascular disease (CVD) and suffered from a median of three symptom categories, most commonly respiratory and neurological symptoms. This cluster also showed a significantly lower forced expiratory volume within 1 s and diffusion capacity of the lung for carbon monoxide. Cluster 3 (18%) was predominantly male (88%) with pre-existing CVD and diabetes. This cluster showed the mildest long COVID, and suffered from symptoms in a median of one symptom category.

**Conclusions:**

Long COVID patients can be clustered into three distinct phenotypes based on their clinical presentation and easily obtainable information. These clusters show distinction in patient characteristics, lung function, long COVID severity and acute SARS-CoV-2 infection severity. This clustering can help in selecting the most beneficial monitoring and/or treatment strategies for patients suffering from long COVID. Follow-up research is needed to reveal the underlying molecular mechanisms implicated in the different phenotypes and determine the efficacy of treatment.

**WHAT IS ALREADY KNOWN ON THIS TOPIC**

- 22%–50% of COVID-19 patients still experience complaints after 4 months.
- Long COVID is a heterogeneous disorder and patients react differently to the same treatment.

**WHAT THIS STUDY ADDS**

- Three distinct long COVID clusters were discovered based on patient characteristics/history, long COVID presentation, acute COVID-19 and lung function. These clusters are described and characterised in detail.

**HOW THIS STUDY MIGHT AFFECT RESEARCH, PRACTICE OR POLICY**

- Clustering based on easily obtainable information and tests could support in selecting the best monitoring and treatment strategy for the individual patients.

## Introduction

Since 2019, over 750 million cases and 6.9 million deaths worldwide are described because of COVID-19 caused by the SARS-CoV-2.^1^ In addition to the impact on physical health, the COVID-19 pandemic has had an enormous impact on mental health and the economy.^2 3^ Infections with SARS-CoV-2 can range from asymptotic to severe, and complaints can include fever, headache, fatigue, cough, pneumonia and dyspnoea.^4^ However, after the initial infection, it is estimated that 22%–50% still suffer from complaints after 4 months.^5^

Just like acute COVID-19, the presentation of patients with long COVID is highly heterogeneous.^6 7^ With these differences in disease manifestations, a one-size-fits-all management plan is not sufficient to treat all patients under the term long COVID. Unsupervised clustering is a way to group entities together with similar features without the need for training labels. Applying clustering to the patient population of long COVID can group together those patients with similar clinical presentation and potentially similar underlying molecular disease pathologies. Consequently, placing patients in one of these clusters would aid in selecting the optimal personalised monitoring and/or treatment strategy for that patient and improve their recovery. Performing high-throughput tests to find those similar molecular mechanisms in a clinical care setting is however not achievable due to the economic costs and manpower required. Thus, there is a need to cluster patients according to their clinical presentation and by using easily performable tests and questionnaires.

Clustering of long COVID patients has been applied previously.^8–11^ However, these studies vary widely in the data used, time since initial infection, number of clusters and cluster characteristics. Mostly, these clusters are based solely on the long COVID symptoms and patient characteristics, sometimes with additional data about the acute infection. In the Precision Medicine for more Oxygen (P4O2) COVID-19 study^12^ we expanded on this information by adding questionnaires about the impact, severity and consequences of the disease and adding lung function tests to non-invasively gain more information. In addition, we performed CT imaging and lab work for cluster interpretation. Finally, we also collected biological samples to complete follow-up experiments and discover the molecular mechanisms underlying the disease phenotypes.

In this study, we aim to perform clustering in the P4O2 COVID-19 cohort to investigate whether patients with long COVID exhibit distinct clinical phenotypes. This is achieved using easily obtainable information, such as clinical presentation, medical history, questionnaires and lung function testing.

## Methods

### Study design and patients

The P4O2 COVID-19 study is a multicentre observational study with the objective of identifying therapeutic biomarkers, personalised medicine or lifestyle interventions for the prevention and treatment of long COVID using a multi-omics approach.^12^ For this study, 95 patients were included in five hospitals across the Netherlands at 3–6 months after SARS-CoV-2 infection. Inclusion criteria were: (1) confirmed SARS-CoV-2 infection, (2) aged 40–65 years, (3) post-COVID-19 outpatient clinic appointment, (4) understanding of the Dutch language and (5) ability to provide informed consent. Patients were excluded if they were terminally ill or involved in another study with investigational or marketed products within 4 weeks prior to study inclusion.

### Patient involvement

Patient representatives have participated during P4O2 consortium meetings to discuss the results and progress of the project and the implications for the patients. Furthermore, the patient advisory board of the department has been regularly updated regarding the status of the project.

### Clinical assessment

During the study visit, patients were asked to provide general information, perform a lung function test and fill in multiple questionnaires. Different demographic and clinical characteristics were collected from the patients or medical records including information such as their sex, age and body mass index (BMI), their medical history regarding asthma, chronic obstructive pulmonary disease (COPD), diabetes and cardiovascular disease (CVD) and finally information about their initial SARS-CoV-2 infection in terms of whether they were hospitalised, hospital duration, oxygen supplementation, WHO severity classification, and whether they suffered from a pulmonary embolism or thrombosis during hospitalisation. The dominant virus type was determined by the SARS-CoV-2 variant most abundant in the Netherlands at the week of the main infection, as determined by the Rijksinstituut voor Volksgezondheid en Milieu.

During this visit, patients also performed a lung function test consisting of spirometry, measurement of the diffusion capacity of the lung for carbon monoxide (DLCO), and underwent a CT scan. Spirometry consisted of the forced expiratory volume within 1 s (FEV1), forced vital capacity (FVC) and the Tiffeneau index (FEV1/FVC). FEV1, FVC and DLCO were used as percent predicted based on sex, BMI, ethnicity and age. For the FEV1 and FVC, the metric was considered abnormal if the percent predicted fell below 90%. For the FEV1/FVC and DLCO, a threshold of 70% (predicted) was used. CT scans were examined by a radiologist in the local hospital.

Questionnaires about the severity of symptoms and impact on daily life were also provided to the patients. These included the Fatigue Severity Scale (FSS),^13^ Patient-Reported Outcomes Measurement Information System (PROMIS),^14^ Primary care PTSD Screen for DSM-5 (PC-PTSD-5),^15^ EuroQoL 5D-5L (EQ5D)^16^ and the Checklist for Cognitive Consequences after an ICU admission (CLC-IC) (adapted version of CLCE-24^17^). In addition, the Utrecht Scale for Evaluation of Revalidation-Participation (USER-P)^18^ and the Hospital Anxiety and Depression Scale (HADS)^19^ questionnaires were administered. Finally, patients were questioned about their symptoms during the first visit and the first monthly questionnaire at home. Complaints were then summarised into the following categories: fatigue, respiratory, gastrointestinal, neurological, cardiovascular and other.

### Statistical analysis

The data used for clustering is summarised in online supplemental table S1. These variables were chosen for their ability to describe the long COVID in a non-invasive manner using easily attainable information and testing. The specific comorbidities chosen to be included due to being either respiratory related, or by having a prevalence such that this variable can contribute to the clustering (≥15 patients). CT scans were not used for clustering due to their radiation risk when applied to a clinical setting, and in this cohort only used for interpretation. The USER-P and HADS questionnaires were not used because of high correlation with other questionnaires and to limit reliance of the clustering on solely questionnaires. The remaining missing data were imputed using Multiple Imputation by Chained Equation as implemented by the mice R package (V.3.14.0).^20^ Unordered categorical variables were imputed using logistic regression, ordered categorical variables were imputed with a proportional odds model and numerical variables were imputed using predictive mean matching. To account for uncertainty due to missing data, 100 different complete imputed data sets were generated for clustering.

10.1136/bmjresp-2023-001907.supp1 Supplementary data

For each imputed data set, the pairwise distance between patients was calculated with the Gower distance for mixed data types. A hierarchical dendrogram was constructed using the Ward.D2 construction method in the *hclust* function from the cluster package (V.2.1.2).^21^ This method was used because of the visual feedback in terms of the cluster separation and deterministic results, in contrast to partitioning around medoids (PAM). Based on a visual inspection of the dendrogram, all dendrograms were divided into three separate clusters. The resulting clustering was saved for each data set, and the Gower distance was applied to get a similarity index over all different clustering solutions. Identical to the individual data sets, a dendrogram was constructed and cut to create three distinct phenotypical clusters. Robustness of the clustering was assessed by applying PAM as a clustering method on the Gower distance in a similar manner as performed above, where similarity between the PAM and hierarchal clusters was determined with the rand index.

Patient characteristics were compared between the different clusters using statistical tests based on the distribution and type of the variable. Categorical variables were examined with a Fisher’s exact test. Numerical variables were assessed using an analysis of variance, if normally distributed by visual inspection, otherwise the Kruskal-Wallis test was used. Post-hoc tests of significant results were performed using pairwise Fisher’s exact tests or pairwise Wilcoxon rank-sum tests. All statistical tests were two-tailed. A Benjamini and Hochberg correction was applied to account for multiple testing, where an adjusted p value below 0.05 was considered statistically significant. All analyses were performed in R (V.4.1.2) using RStudio (V.2021.09.1+372).^22^

## Results

### P4O2 COVID-19 cohort can be divided into three phenotypically similar clusters

**Figure 1:**
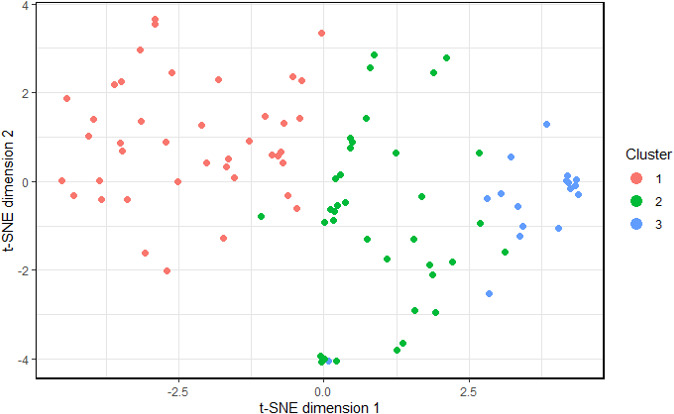
t-SNE plot showing the separation of the clusters projected on a 2-dimensional space.

The patient characteristics can be found in table 1. The patient population had an even distribution with regards to sex (49.5% female), had an average age of 54.1 (SD=6.2) years and was mostly overweight or obese with an average BMI of 30.5 (SD=5.3). The most common symptom categories after 3–6 months included respiratory symptoms (78.9%), neurological symptoms (70.5%) and fatigue (69.5%). Based on visual inspection of dendrograms from individual imputed datasets (online supplemental figure S1), the 95 patients from the P4O2 COVID-19 cohort were divided into three clusters of 42, 36 and 17 patients. Cluster separation can be seen in a t-SNE plot in figure 1.

**Table 1:** Baseline characteristics of the P4O2 COVID-19 cohort

	All patients (N=95)
General characteristics	
Sex (female)	47/95 (49.5%)
Age (years)	54.1±6.2
BMI (kg/m^2^)	n=94; 30.5±5.3
Comorbidities	
Asthma	16/94 (17.0%)
COPD	6/94 (6.4%)
CVD	27/93 (29.0%)
Diabetes	15/94 (16.0%)
Symptom categories	
Fatigue	66/95 (69.5%)
Respiratory	75/95 (78.9%)
Neurological	67/95 (70.5%)
Cardiovascular	25/95 (26.3%)
Gastrointestinal	29/95 (30.5%)
Other	18/95 (18.9%)
Lung function	
FEV1 % pred	n=90; 91.5±17.1
FVC % pred	n=90; 89.8±18.1
FEV1/FVC	n=90; 80.1±8.0
DLCO % pred	n=89; 79.9±19.9
Questionnaires	
FSS	n=87; 5.6 (4.2, 6.3)
PROMIS	n=82; 28.7±8.0
PC-PTSD-5	n=83; 1.0 (0.0, 2.0)
EQ5D	n=83; 9.0 (6.0, 11.0)
CLC-IC	n=80; 5.0 (2.0, 8.0)
USER-P	n=83; 80.0 (60.0, 96.8)
HADS Depression	n=78; 3.5 (1.0, 7.8)
HADS Anxiety	n=80; 4.0 (1.0, 8.0)
Acute phase WHO severity	
Mild	10/95 (10.5%)
Moderate	61/95 (64.2%)
Severe	24/95 (25.3%)
Acute phase duration/complications	
Hospital duration	n=84; 8.0 (5.0, 15.2)
Pulmonary embolism	15/92 (16.3%)
Thrombosis	14/91 (15.4%)
Dominant virus type	
Alpha	43/95 (45.3%)
Delta	41/95 (43.2%)
Omicron	11/95 (11.6%)
CT abnormalities	
Ground-glass opacity/consolidations	54/87 (62.1%)
Bronchiectasis	19/87 (21.8%)
Subpleural reticulation	23/87 (26.4%)
Honeycombing	2/87 (2.3%)
Lymphadenopathy	9/87 (10.3%)
Airtrapping	10/87 (11.5%)
Vaccination	
No	28/95 (29.5%)
Yes, 1 or more doses	67/95 (70.5%)
Smoking status	
Never smoker	40/95 (42.1%)
Ex-smoker	51/95 (53.7%)
Current smoker	4/95 (4.2%)
Level of education	
Secondary education	19/79 (24.1%)
Vocational education	33/79 (41.8%)
Bachelor	19/79 (24.1%)
Master	8/79 (10.1%)

For numerical values, normally distributed data is given as mean±SD, otherwise as median (IQR). Categorical variables are displayed as n (%). When not all data is available, the number of patients with data available is given.

BMI, body mass index; CLC-IC, Checklist for Cognitive Consequences after an ICU Admission; COPD, chronic obstructive pulmonary disease; CVD, cardiovascular disease; DLCO, diffusion capacity of the lungs for carbon monoxide; EQ5D, EuroQoL 5D-5L; FEV1, forced expiratory volume in 1 s; FSS, Fatigue Severity Scale; FVC, forced vital capaticy; HADS, Hospital Anxiety and Depression Scale; PC-PTSD-5, Primary Care PTSD Screen for DSM-5; PROMIS, Patient-Reported Outcomes Measurement Information System; USER-P, Utrecht Scale for Evaluation of Revalidation-Participation.

### Clusters showed differences in sex and patient comorbidities

The three clusters showed a similar distribution in age (table 2). However, there was an imbalance in the distribution of sex in the clusters. Cluster 1 contained predominantly female patients (92.9%), while the other two clusters contained predominantly males (83.3% and 88.2%). Patients from cluster 1 had a slightly higher BMI compared with the other clusters. However, this failed to reach statistical significance (31.7 compared with 29.9 and 28.5, p=0.13). Patients in cluster 1 also showed a significantly higher rate of asthma (29.3%) compared with cluster 2 (11.1%) and 3 (0.0%), while also showing lower rates of CVD (12.5% compared with 41.7% and 41.2%). Cluster 3 contained relatively more patients with pre-existing diabetes with 47.1%, which was significantly more than 2.4% in cluster 1 and 16.7% in cluster 2. In the entire cohort, these comorbidities were slightly correlated with sex (asthma: p=0.03, CVD: p=0.07, diabetes: p=0.09).

**Table 2:** Baseline characteristics of the P4O2 COVID-19 cohort with cluster separation

	Cluster 1 (N=42)	Cluster 2 (N=36)	Cluster 3 (N=17)	Adj p value
General characteristics				
Sex (female)	39/42 (92.9%)	6/36 (16.7%)	2/17 (11.8%)	**<0.001**
Age (years)	53.6±6.2	54.7±6.3	54.2±6.1	0.799
BMI (kg/m^2^)	31.7±5.8	29.9±5.1	n=16; 28.5±3.0	0.133
Comorbidities				
Asthma	12/41 (29.3%)	4/36 (11.1%)	0/17 (0.0%)	**0.025**
COPD	4/41 (9.8%)	2/36 (5.6%)	0/17 (0.0%)	0.640
CVD	5/40 (12.5%)	15/36 (41.7%)	7/17 (41.2%)	**0.018**
Diabetes	1/41 (2.4%)	6/36 (16.7%)	8/17 (47.1%)	**<0.001**
Symptom categories				
Fatigue	41/42 (97.6%)	23/36 (63.9%)	2/17 (11.8%)	**<0.001**
Respiratory	41/42 (97.6%)	31/36 (86.1%)	3/17 (17.6%)	**<0.001**
Neurological	34/42 (81.0%)	27/36 (75.0%)	6/17 (35.3%)	**0.008**
Cardiovascular	16/42 (38.1%)	8/36 (22.2%)	1/17 (5.9%)	0.056
Gastrointestinal	21/42 (50.0%)	5/36 (13.9%)	3/17 (17.6%)	**0.004**
Other	12/42 (28.6%)	4/36 (11.1%)	2/17 (11.8%)	0.217
Lung function				
FEV1 % pred	n=40; 96.2±14.8	n=35; 84.7±18.7	n=15; 94.7±14.6	**0.022**
FVC % pred	n=40; 94.6±17.4	n=35; 83.5±18.9	n=15; 91.9±13.9	0.052
FEV1/FVC	n=40; 80.7±7.9	n=35; 79.5±9.3	n=15; 80.0±4.9	0.811
DLCO % pred	n=40; 85.9±17.0	n=35; 69.3±20.0	n=14; 89.7±15.8	**<0.001**
Questionnaires				
FSS (↓)	n=39; 6.0 (5.1, 6.6)	n=33; 5.6 (5.1, 6.2)	n=15; 2.2 (1.6, 3.1)	**<0.001**
PROMIS (↑)	n=39; 26.7±7.2	n=28; 25.8±6.3	n=15; 39.5±1.3	**<0.001**
PC-PTSD-5 (↓)	n=39; 1.0 (0.0, 3.0)	n=29; 1.0 (0.0, 1.0)	n=15; 0.0 (0.0, 0.0)	**0.003**
EQ5D (↓)	n=40; 11.0 (8.0, 12.2)	n=28; 9.5 (7.8, 11.0)	n=15; 5.0 (5.0, 6.0)	**<0.001**
CLC-IC (↓)	n=37; 6.0 (3.0, 8.0)	n=29; 5.0 (3.0, 9.0)	n=14; 0.5 (0.0, 1.0)	**<0.001**
USER-P (↑)	n=39; 73.3 (56.4, 88.9)	n=29; 72.7 (53.3, 83.3)	n=15; 100.0 (100.0, 100.0)	**<0.001**
HADS Depression (↓)	n=35; 4.0 (2.0, 8.5)	n=28; 5.0 (2.8, 9.0)	n=15; 0.0 (0.0, 1.0)	**<0.001**
HADS Anxiety (↓)	n=36; 5.5 (1.0, 10.0)	n=29; 3.0 (2.0, 8.0)	n=15; 2.0 (1.0, 4.5)	0.273
Acute phase WHO severity				
Mild	9/42 (21.4%)	1/36 (2.8%)	0/17 (0.0%)	0.063
Moderate	26/42 (61.9%)	24/36 (66.7%)	11/17 (64.7%)	
Severe	7/42 (16.7%)	11/36 (30.6%)	6/17 (35.3%)	
Acute phase duration/complications				
Hospital duration	n=33; 8.0 (4.0, 11.0)	n=34; 7.5 (5.2, 27.5)	7.0 (3.0, 12.0)	0.642
Pulmonary embolism	5/40 (12.5%)	9/35 (25.7%)	1/17 (5.9%)	0.249
Thrombosis	3/40 (7.5%)	7/34 (20.6%)	4/17 (23.5%)	0.249
Dominant virus type				
Alpha	17/42 (40.5%)	18/36 (50.0%)	8/17 (47.1%)	0.808
Delta	20/42 (47.6%)	13/36 (36.1%)	8/17 (47.1%)	
Omicron	5/42 (11.9%)	5/36 (13.9%)	1/17 (5.9%)	
CT abnormalities				
Ground-glass opacity/consolidations	22/38 (57.9%)	23/33 (69.7%)	9/16 (56.2%)	0.622
Bronchiectasis	6/38 (15.8%)	8/33 (24.2%)	5/16 (31.2%)	0.496
Subpleural reticulation	7/38 (18.4%)	10/33 (30.3%)	6/16 (37.5%)	0.347
Honeycombing	0/38 (0.0%)	2/33 (6.1%)	0/16 (0.0%)	0.395
Lymphadenopathy	3/38 (7.9%)	4/33 (12.1%)	2/16 (12.5%)	0.787
Airtrapping	9/38 (23.7%)	0/33 (0.0%)	1/16 (6.2%)	**0.013**
SARS-CoV-2 vaccination				
No	14/42 (33.3%)	6/36 (16.7%)	8/17 (47.1%)	0.249
Yes, 1 or more doses	28/42 (66.7%)	30/36 (83.3%)	9/17 (52.9%)	
Smoking status				
Never smoker	15/42 (35.7%)	16/36 (44.4%)	9/17 (52.9%)	0.139
Ex-smoker	27/42 (64.3%)	16/36 (44.4%)	8/17 (47.1%)	
Current smoker	0/42 (0.0%)	4/36 (11.1%)	0/17 (0.0%)	
Level of education				
Secondary education	7/39 (17.9%)	8/26 (30.8%)	4/14 (28.6%)	0.102
Vocational education	18/39 (46.2%)	11/26 (42.3%)	4/14 (28.6%)	
Bachelor	13/39 (33.3%)	3/26 (11.5%)	3/14 (21.4%)	
Master	1/39 (2.6%)	4/26 (15.4%)	3/14 (21.4%)	

P values were calculated using the analysis of variance or Kruskal-Wallis tests for numerical data, and a Fisher’s exact test for categorial data. Data are shown as mean±SD or median (IQR) for numerical data and as n (%) for categorical data. When not all data are available, the number of patients with data available is given. For each questionnaire it is indicated with an arrow if a higher or lower score is considered better. P values below 0.05 are highlighted in bold. Adj p value: adjusted p value (Benjamini and Hochberg).

BMI, body mass index; CLC-IC, Checklist for Cognitive Consequences after an ICU Admission; COPD, chronic obstructive pulmonary disease; CVD, cardiovascular disease; DLCO, diffusion capacity of the lungs for carbon monoxide; EQED, EuroQoL 5D-5L; FEV1, forced expiratory volume in 1 s; FSS, Fatigue Severity Scale; FVC, forced vital capaticy; HADS, Hospital Anxiety and Depression Scale; PC-PTSD-5, Primary Care PTSD Screen for DSM-5; PROMIS, Patient-Reported Outcomes Measurement Information System; USER-P, Utrecht Scale for Evaluation of Revalidation-Participation.

### Clusters showed differences in the number of symptom categories per patient, lung function and long COVID severity

**Figure 2:**
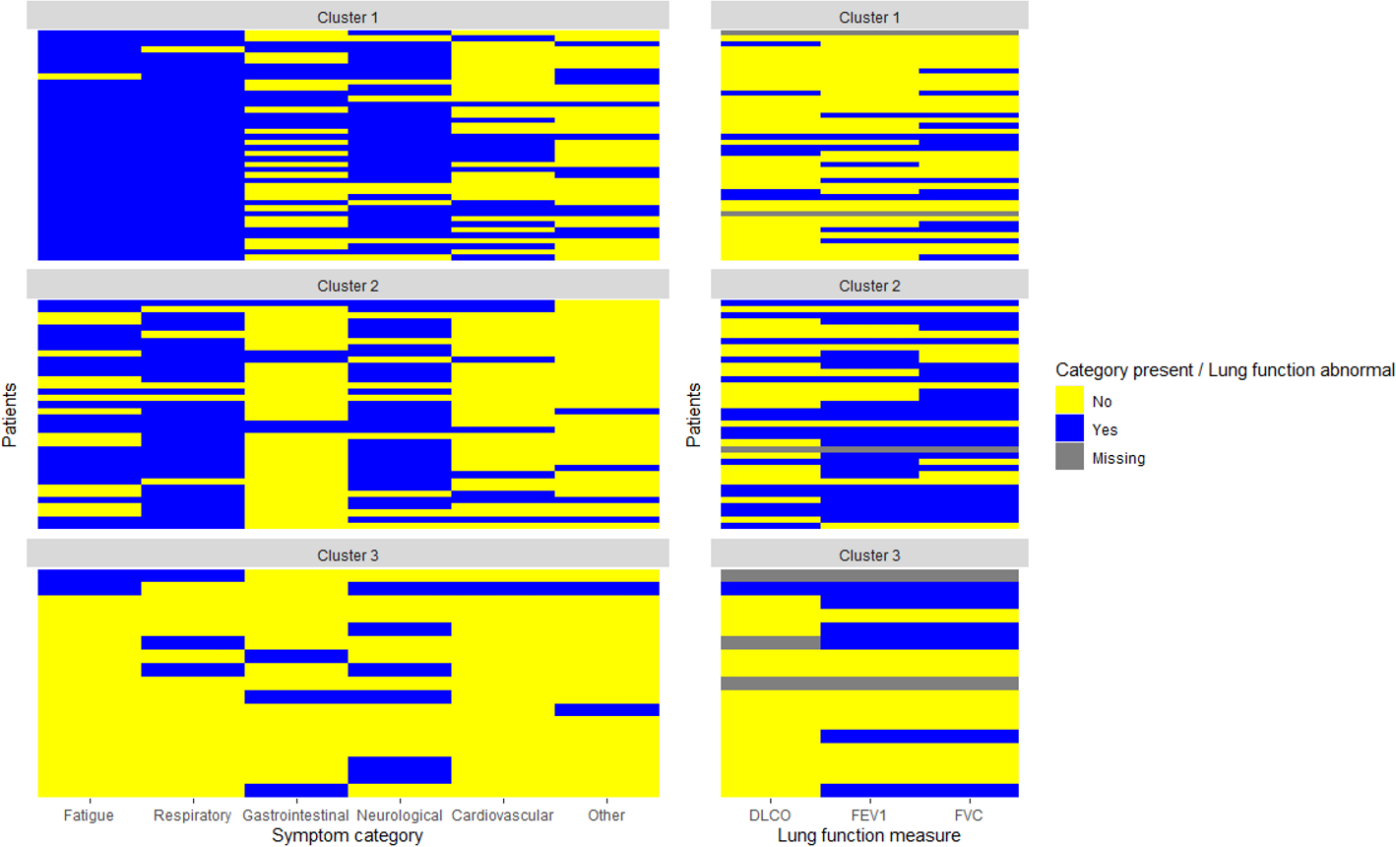
Heatmap depicting the presence or absence of rest complaints in the symptom categories or lung function, divided into the clusters constructed in this study. This heatmap shows that patients in cluster 2 suffer from relatively more symptom categories, while patients in cluster 4 suffer from relatively few symptom categories. In addition, cluster 3 shows relatively more abnormalities in lung function compared with other clusters.

The symptom categories per patient in each cluster are summarised in figure 2. This figure shows that patients in cluster 1 suffered from relatively more symptoms over different categories (median of 4 symptom categories), while patients in cluster 2 suffered from symptoms in a median of 3 categories. Finally, patients in cluster 3 experienced symptoms from a median of 1 category. Patients in cluster 1 showed predominantly fatigue (97.6%) and respiratory symptoms (97.6%), additionally, these patients also showed a high rate of neurological (81.0%) and gastrointestinal symptoms (50%); in cluster 2 patients suffered mostly from respiratory (86.1%) and neurological symptoms (75%) and fatigue (63.9%); patients in cluster 3 showed mostly neurological symptoms (35.3%). In terms of lung function, we found a significantly reduced FEV1 and DLCO for patients in cluster 2, while the FVC just slightly failed to reach statistical significance (p=0.052).

Based on the questionnaire results, cluster 3 scored significantly better in terms of fatigue (FSS), physical, mental and social well-being (PROMIS), self-care (EQ5D), cognitive consequences after an ICU admission (CLC-IC), participation (USER-P) and depression (HADS) compared with both other clusters.

### No significant differences separating the clusters in other variables

We were unable to find differences between the clusters during the acute phase of SARS-CoV-2 infection. However, 9 out of 10 patients that were not hospitalised during this phase were all placed in cluster 1. For the WHO severity classifications this however lacked the power to give a statistically significant result (p=0.063). No statistically significant differences between the clusters were found in terms of the dominant virus type at the time of infection, vaccination status, smoking status and education level. In terms of rest abnormalities found on CT-imaging, airtrapping was significantly more common in cluster 1.

### Clusters showed moderate stability in regards to clustering method

When PAM clustering was applied to the Gower distance instead of hierarchal clustering, the different clustering methods showed a rand index of 0.62. While patients in the second and third clusters often stay in the same cluster they already belonged, patients from the hierarchal cluster 1 were more distributed over PAM cluster 1 and 2. This is also visible in online supplemental figure S2 depicting a t-SNE plot of this cluster separation. Hierarchal clustering is a better fit to this data, showing more defined clusters and better cluster separation than PAM clustering.

## Discussion

In this study, we aimed to cluster the patients suffering from long COVID in the P4O2 COVID-19 cohort into similar clinical phenotypes using easily obtainable information. Cluster 1 consisted of predominantly females with slightly higher BMI and pre-existing asthma. They suffered from complaints in a median of four symptom categories, with most commonly fatigue and respiratory and neurological symptoms. Patients in this cluster also showed a milder acute SARS-CoV-2 infection, and showed signs of airtrapping on a CT scan more often. Patients in cluster 2 were predominantly male with pre-existing CVD. They suffered from a median of three symptom categories, with most commonly fatigue and respiratory symptoms. They also showed a significantly reduced FEV1 and DLCO. Patients in cluster 3 were also predominantly male with pre-existing CVD and diabetes. They showed significantly fewer symptoms, with having a median of one symptom category. They also scored significantly better on nearly all questionnaires.

In our clustering, we found that cluster 1 was predominantly female with severe long COVID, however, with a milder acute infection compared with the other clusters. Research has indeed shown that being male is a risk factor for severe COVID-19,^23^ while risk factors for long COVID include being female.^24^ This same pattern was also discovered in other clustering efforts.^8–10^ In addition, it has also been reported that pre-existing asthma is a risk factor for developing long COVID.^24^ We already found that in our cohort, 17% of patients suffer from asthma, this is higher than the prevalence of asthma in the Dutch population at 6%.^25^ In the most severe cluster, asthma was even more common at 29% of patients, suggesting a potential link between asthma and long COVID severity, potentially mediated by a reduction in asthma control after SARS-CoV-2 infection.^26 27^

Several factors involved in the severity of acute COVID-19 were not found statistically different between the clusters. These include: vaccination status, smoking status and the SARS-CoV-2 virus type. In literature there has been conflicting information about the impact of vaccination of long COVID, with studies showing no impact of vaccination on the development of long COVID, while other studies showed a reduced risk of long COVID after vaccination.^28^ In addition, getting vaccinated after already developing long COVID also showed no influence on long COVID severity, as found in a study where 17% of patients showed a decrease in severity, while in 21% the severity increased.^29^ We were also not able to draw any conclusions about the impact of smoking on the clustering, this is potentially a problem regarding power, as there are only four current smokers in our cohort. Many patients in our cohort were former smokers, with the times since they quit ranging from 11 to 480 months. Not being able to make conclusions about the dominant virus type is also a result of low power. Research has shown that patients infected with the omicron variant are less likely to develop long COVID, and suffer from fewer symptoms compared with those infected with the delta variant.^30^ We did not see enough with either virus type to obtain enough power to make these conclusions.

This study was not the first to perform clustering on a long COVID patient population. Similarly to other clustering efforts,^8 10 11^ we found that three clusters best describe the long COVID population. Equally, these studies all distinguish a cluster that showed fewer symptoms compared with the other clusters and contains more males. Interestingly though, this same cluster is marked by having fewer comorbidities than the other clusters in.^10 11^ In our mild cluster this was not a defining feature, with diabetes even being the most common comorbidity in this cluster. There has been conflicting literature about the relation between diabetes and long COVID,^31^ however, there does seem to be evidence that diabetes increases the risk for developing long COVID. One reason for the discrepancy between the comorbidities and cluster severity might be the correlation between comorbidities and sex in our cluster. As our clusters are heavily dependent on sex, this might have influenced the distributions of comorbidities as well. Other studies showed the distinction between the other clusters either based on further severity or several symptoms, however, we found an important difference between cluster 1 and 2 to be in the lung function instead, where cluster 2 showed a markedly lower lung function compared with clusters 1 and 3. The other studies did also show hints for a cluster with a more respiratory axis at 3 months after infection. A larger study with only two clusters found shortness of breath more prevalent in one their clusters,^32^ while in Fischer *et al*,^11^ shortness of breath was significantly more common in their moderate severity cluster compared with the severe cluster. However, none of these studies confirmed this further with pulmonary function testing.

Besides the cluster of respiratory complaints and low lung function in cluster 2 and gastrointestinal complaints in cluster 1, we were not able to describe clusters based on particular symptom patterns besides quantity. One reason for this could be the classification of symptoms. Here the symptoms were grouped into systemic categories instead of using the presence of each symptom. This has both advantages and disadvantages. While we do lose the patterns of symptoms within each category, our method allowed the clustering to not be dominated by many symptoms from a single category. In addition, while a systematic approach like in Reese *et al* 9 helps describing each person, it will result in sparse data which complicates clustering. Our method does not require specific symptoms to be questioned and can be applied more easily to other populations.

The strength of this study lies in the scope of information that we collected. This allowed us to view the patients in more detail, such as lung function and CT scans. Taking lung function into account distinguished between cluster 1 and cluster 2. However, due to the resources we require from patients, our sample size is relatively small compared with other long COVID clustering studies.^8–11^ Because of this, we did not have enough power to make conclusions about vaccination status, CT abnormalities besides airtrapping and level of education, where we do see potential differences between the clusters. Due to the nature of the study, we do not have information from before the SARS-CoV-2 infection. Consequently, we did not know whether lung function or radiological abnormalities were already present, potentially providing a bias for our clustering (lung function) or interpretation of the clustering.

Long COVID is a heterogeneous disease and here we clustered those patients into phenotypically similar clusters based on information that is easily obtainable from the patient characteristics, medical history, clinical presentation, questionnaires and non-invasive tests. We discovered clusters that differ in severity of initial SARS-CoV-2 infection, long COVID characteristics and symptoms, sex distribution, lung function and comorbidities. These clusters using easily obtainable information in a clinical setting could help differentiate patients into groups with similar underlying disease and can help optimise treatments for the individual patient. However, to get to this point, more research is needed to find underlying molecular pathologies for each cluster, and the efficacy of treatments has to be established for each cluster. In long COVID patients with pulmonary function abnormalities, pulmonary habilitation has been shown to increase lung function and quality of life, while also decreasing symptoms of fatigue and dyspnoea.^33^ That treatment might be of particular interest for patients in cluster 2, which showed lung function abnormalities. This study provides a start to use the information about the patients to research underlying pathways and with that knowledge select the best monitoring and/or treatment strategy for a personalised medicine approach in long COVID.

10.1136/bmjresp-2023-001907.supp2 Supplementary data

## Data Availability

Data are available upon reasonable request. The data are not publicly available due to agreements made by the consortium, that only allow access by each consortium partner to specific data that answers their prespecified research questions. A request for access to data by organisations outside of the consortium can be submitted to the P4O2 Data Committee (via p4o2@amsterdamumc.nl) and the research will need to be performed in collaboration with one of the P4O2 consortium partners.
