# Supplementary material for: Long COVID exhibits clinically distinct phenotypes at 3–6 months post-SARS-CoV-2 infection: results from the P4O2 consortium
Source: BMJ Open Respir Res. 2024 Apr 24;11(1):e001907. doi: 10.1136/bmjresp-2023-001907 (PMC11043734; doi:10.1136/bmjresp-2023-001907)
Supplement: Supplementary data [file bmjresp-2023-001907supp001.pdf]

Supplementary Tables

Table S1: All variables that were used to create the clusters.

Patient characteristics/co-morbidities	Questionnaires	Lung function	Long COVID symptom categories	Acute phase characteristics
BMI<br>Age<br>Sex<br>Asthma<br>COPD<br>CVD<br>Diabetes	FSS<br>PROMIS<br>PC-PTSD-5<br>EQ5D<br>CLCIC	FEV1<br>FVC<br>FEV1/FVC<br>DLCO	Fatigue<br>Respiratory<br>Neurological<br>Cardiovascular<br>Gastrointestinal<br>Other	Oxygen received (type)<br>Hospitalized<br>Hospital duration<br>Thrombosis<br>Pulmonary embolism

BMI: body mass index, COPD: chronic obstructive pulmonary disease, CVD: cardiovascular disease, FEV1: forced expiratory volume, FVC: forced vital capacity, DLCO: diffusion capacity of the lungs for carbon monoxide.

Supplementary Figures

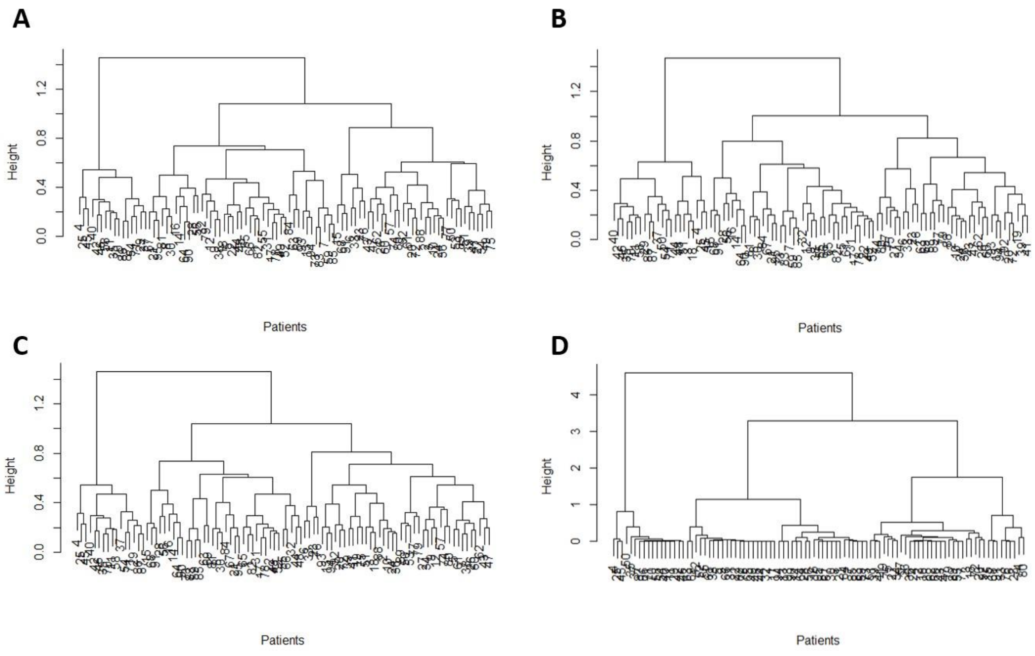

Figure S1: Dendrograms from hierarchal clustering of individual imputed data sets (A-C) and the dendrogram from the consensus clustering (D).

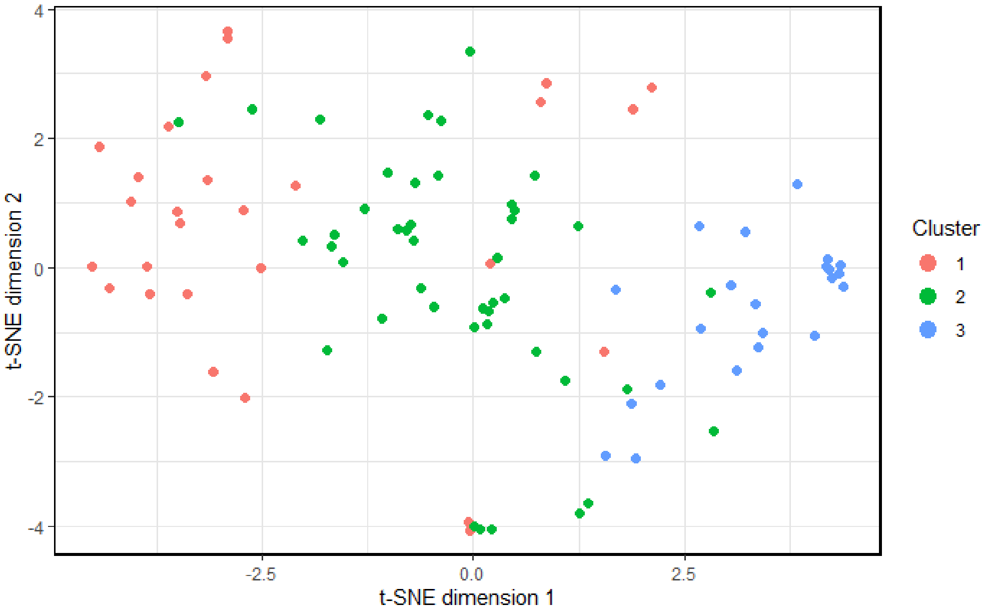

Figure S2: t-SNE plot showing the separation of the clusters created by the PAM clustering method projected on a 2-Dimensional space.
